# Physician‒Scientists in Italian Dermatology: Hurdles and Perspectives

**DOI:** 10.1016/j.xjidi.2021.100077

**Published:** 2021-11-25

**Authors:** Carlo Pincelli, Antonio Costanzo

**Affiliations:** 1 Laboratory of Cutaneous Biology, Section of Dermatology, Department of Surgical, Medical, Dental and Morphological Sciences, University of Modena and Reggio Emilia, Modena, Italy; 2 Dermatology Unit, Department of Biomedical Sciences, Humanitas University, Rozzano-Milan, Italy

## Abstract

Physicians, including dermatologists, with expertise in clinical and basic research, play a pivotal role in the advancement of medical science. Although the number of residents in dermatology has been increasing and our specialty is among the most requested in Italy, the disaffection of young dermatologists for research is a chronic and apparently irreversible trend. This commentary analyzes the reasons and suggests some ideas to counteract this alarming tendency.

### Physicians‒scientists’ essential role in biomedicine

Physician‒scientists (clinician‒scientists) are medical doctors who have the great privilege and the opportunity to see patients and carry out basic and/or clinical research at the same time. Physician‒scientists have definitely the broadest vision of biomedicine, which allows them to better understand diseases and the underlying mechanisms. Clinicians who devote themselves to research are essential for the progress of medicine because their bench-to-bedside activity is key for making new discoveries, for example, finding new disease targets and potential innovative diagnostic tools and therapies. Indeed, in the past 100 years, physician‒scientists in general medicine and dermatology have contributed to a large extent to a number of pioneering inventions. It is interesting to note that approximately one third of the Nobel Prize winners in Medicine are physician‒scientists, whereas about 70% of chief scientific officers at most important big pharmaceuticals are medical doctors (Jain et al., 2019). Clinician‒scientists in dermatology have been playing a critical role in understanding the pathogenesis of dermatologic diseases through pivotal research related to molecular biology, immunology, biochemistry, and gene therapy, etc. This is witnessed by the huge number of high-impact publications in cutaneous biology from groups led by physician‒scientists.

### Clinician‒scientists in dermatology are an endangered species

Dermatology seems to be very attractive for medical doctors because the number of applications and actual residents has been increasing over recent decades. In Italy, dermatology ranked first or second among the most requested residency specialties in the last 3 years. In addition, skin is a very easy-to-access organ that, more than other specialties, should stimulate the curiosity and appeal for a better understanding of the disease processes. Dermatologists would be in the best position to enter a research career path and become physician‒scientists.

Against this, it appears that dermatologists willing to become investigators in cutaneous biology have become a kind of endangered species not only in the United States (Li et al., 2021) but also all over the world. In most countries, there has been a marked reduction in the number of dermatologists interested in entering a research path, whereas in the United States, only half of MD/PhD dermatologists are engaged in investigational activities (Andriole et al., 2021).

### Italian dermatologists and research: a complicated relationship

In this respect, Italy is certainly not an exception. Despite the lack of reliable figures on this topic in Italy, it is easy to state that in our country, the disaffection of dermatologists from research is a chronic issue. Unfortunately, Italian dermatology has always regarded research as a kind of hobby for eccentric doctors who spend too much time in the laboratory, neglecting patients. Old-generation chairs and, with few exceptions, also younger chiefs of departments demand that their students, young doctors, and specialists follow a career path under their strict supervision, not allowing them to do any research, to go abroad, and to spend time in research institutions. Of course, this is the average situation, and over the years, a bunch of passionate dermatologists, including the authors of this commentary, have managed to work full time with patients and find the way of going to the laboratory, maybe alone or with the support of some technician willing to help and, in many instances, sharing with them the same enthusiasm. Believe it or not, this negligible number of dermatologists has contributed to a discrete number of investigations that have resulted in seminal publications in high-impact journals. Yet, the willingness of few dermatologists and their example over the past 30 years has failed to change the mentality of young dermatologists who refuse to enter an academic/research career. However, it should be emphasized that nothing in Italy favors an inversion of this trend because of a number of reasons as follows:

1. Only two to three departments of dermatology in Italy have an up and running laboratory.
2. Residents are employees of the National Health Service and are overwhelmed by clinical activities.
3. There are no actual PhD programs for clinician‒scientists in dermatology tailored on what would be important for them to be stimulated to enter a research path. Eventually, the vast majority of MD/PhD dermatologists end up going back to clinical work.
4. The reluctance to pursue a research career is even more pronounced among dermatologists than among other specialties because of the opportunities young dermatologists have to devote to cosmetic dermatology and earn a lot of money in no time, as opposed to a poor career in skin biology.
5. No funds or other facilitations would be provided for the few residents who, despite the hurdles, are still willing to commit to a dual-career track.

On top of these, we observe a worrying lack of a culture that has characterized the Italian dermatologic establishment and its chairs for the past 50 years.

### Perspectives and potential solutions

We strongly believe that in Italy, a new culture that favors research at all levels is only possible with the initiative and support of the department chairs, who can promote a supportive departmental environment. In this respect, it is encouraging to note that over the past 10 years, a new generation of young chairs has been appointed. A few of them seem to have a new mentality and should be concerned about the decline of dermatology. It is a fact that without research, that is, without publications in high-impact journals and consequent increase in reputation, in Italy, dermatology will become less and less respected not only by the scientific environment but mostly also by healthcare providers and politicians who will see our specialty as a minor one with severe consequences for our patients.

The role of department chairs in Italy will be key to the formation of physician‒scientists in dermatology. Although it is not an easy task, they have the responsibility of setting up the conditions and the environment for allowing young doctors and residents to carry out research while taking care of patients. First of all, chairs must provide protected research time, laboratory space, funds, and some help from technical personnel for those who are really attracted by research and are talented for a dual-career path. Yet, here comes the issue: how to identify young doctors and residents and have them involved and interested in investigative dermatology? To this purpose, mentorship, which is currently lacking in Italy, is critical. Chairs must be supported by associates and younger colleagues who should be scouting young and passionate doctors and start mentoring them as early as possible. This can be done through frequent seminars and discussions with basic scientists about the potential mechanisms underlying skin diseases. In addition, mentees should be involved in clinical trials with novel drugs that are more and more numerous. This should stimulate their curiosity about the mechanism of action and drive them to the research path.

It is true that this cannot be achieved by the goodwill of a single chair. In contrast, the example of a few can trigger a virtuous circle. The fascinating story of Irwin Freedberg and his predecessor Marion Sulzberger who, many years ago, at New York University managed to create a physician‒scientist‒friendly faculty with a perfect balance between clinical and research activities should be inspiring young Italian chairs (Chu and Orlow, 2006). In any case, we strongly believe that at least a group of visionary chairs should build a network that will, possibly by interacting with other specialties, discuss with the Ministry of Health and the Ministry of Innovation, University and Research to reshape the residency programs and to create new ones that take into higher account the importance of forming physician‒scientists who are indispensable for the future of dermatology and medicine in general.

An incentive to involve more students and residents in a research path could be to teach them something either medical schools or dermatology residency programs do not offer, that is, the process of drug development from the preclinical to the clinical phases, for them to become interested in the mechanism of action of drugs and the way to bring them to the market. This is particularly relevant in these very years with the continuous and enormous release of new drugs and the huge increase in clinical trials. This should stimulate their curiosity and make them understand that research is essential to have these innovative drugs at our disposal. Having a research background would allow them not to simply act as the doctor who prescribes medicines but to be proactive in discussing with pharmaceuticals and advising them. In addition, their perspective would be to have the opportunity someday to work in the pharmaceutical industry and turn their poor career into a very rewarding activity. Physician‒scientists with expertise in both biology and clinical medicine have by far the best attitude to invent new drugs.

Although the situation is different, at least in Europe, from country to country, and some of these keep forming good physicians‒scientists, resulting by the way in better research productivity, the distancing of residents and young dermatologists from research is certainly a common trend. This is why European countries should take joint initiatives to face and solve this critical issue. For example, the European Union commission funds different kinds of teaching and educational programs. We strongly believe that key opinion leaders and chiefs of departments are very much concerned about this issue. Thus, they should realize the urgency of it and take action now.

## ORCID

Carlo Pincelli: http://orcid.org/0000-0003-4416-2637

## Conflict of Interest

The authors state no conflict of interest
